# HIV-1 subtype C envelope characteristics associated with divergent rates of chronic disease progression

**DOI:** 10.1186/1742-4690-7-92

**Published:** 2010-11-04

**Authors:** Derseree Archary, Michelle L Gordon, Taryn N Green, Hoosen M Coovadia, Philip JR Goulder, Thumbi Ndung'u

**Affiliations:** 1 HIV Pathogenesis Programme, Doris Duke Medical Research Institute, Nelson R. Mandela School of Medicine, University of KwaZulu-Natal, Durban, South Africa; 2 Department of Pediatrics, Nuffield Department of Medicine, The Peter Medawar Building for Pathogen Research, Oxford University, Oxford, UK

## Abstract

**Background:**

HIV-1 envelope diversity remains a significant challenge for the development of an efficacious vaccine. The evolutionary forces that shape the diversity of envelope are incompletely understood. HIV-1 subtype C envelope in particular shows significant differences and unique characteristics compared to its subtype B counterpart. Here we applied the single genome sequencing strategy of plasma derived virus from a cohort of therapy naïve chronically infected individuals in order to study diversity, divergence patterns and envelope characteristics across the entire HIV-1 subtype C gp160 in 4 slow progressors and 4 progressors over an average of 19.5 months.

**Results:**

Sequence analysis indicated that intra-patient nucleotide diversity within the entire envelope was higher in slow progressors, but did not reach statistical significance (p = 0.07). However, intra-patient nucleotide diversity was significantly higher in slow progressors compared to progressors in the C2 (p = 0.0006), V3 (p = 0.01) and C3 (p = 0.005) regions. Increased amino acid length and fewer potential N-linked glycosylation sites (PNGs) were observed in the V1-V4 in slow progressors compared to progressors (p = 0.009 and p = 0.02 respectively). Similarly, gp41 in the progressors was significantly longer and had fewer PNGs compared to slow progressors (p = 0.02 and p = 0.02 respectively). Positive selection hotspots mapped mainly to V1, C3, V4, C4 and gp41 in slow progressors, whereas hotspots mapped mainly to gp41 in progressors. Signature consensus sequence differences between the groups occurred mainly in gp41.

**Conclusions:**

These data suggest that separate regions of envelope are under differential selective forces, and that envelope evolution differs based on disease course. Differences between slow progressors and progressors may reflect differences in immunological pressure and immune evasion mechanisms. These data also indicate that the pattern of envelope evolution is an important correlate of disease progression in chronic HIV-1 subtype C infection.

## Background

The rate of disease progression in HIV-1 infected individuals is determined by a complex interplay of viral characteristics, host genetic factors, immune responses and environmental factors. The high viral replication rate, the lack of proof-reading mechanism by the HIV reverse transcriptase enzyme, and high recombination rate are characteristics that ensure that the virus continuously mutates and evolves, resulting in both HIV diversification and viral escape from host immune responses [1,2]. Viral diversity and the constant generation of new viral quasispecies that may not be recognized or eliminated by the host immune mechanisms, particularly contemporaneous virus-specific cytotoxic CD8+ T-cells or neutralizing antibodies, are major impediments for the development of an efficacious HIV-1 vaccine [3,4].

The HIV-1 envelope (Env) subunits gp120 and gp41 are the only viral proteins that are exposed on the virus surface, and they are under continuous host selective pressure, as they are key determinants of the target host cell range and are important targets of neutralizing antibodies and CD8 T cell responses. Specific Env sequence characteristics such as the overall amino acid diversity, the number of putative N-linked glycosylation sites (PNGs), and the length of variable loops have been shown to influence or correlate with antibody neutralization sensitivity, cell tropism, co-receptor utilization and virus transmission [5-7]. Studies of Env diversity can also provide important clues for selective forces that may significantly influence the rate of disease progression or alternatively identify specific regions of the Env protein that comprise important targets of effective immune pressure which may be important considerations in rational HIV-1 vaccine design.

In HIV-1 subtype B, the relationship between HIV-1 Env diversity and disease progression is complex, as illustrated by a series of studies. In one early study, HIV-1 Env hypervariable region 3 (V3 loop) diversity was shown to increase with time [8]. A subsequent study showed that Env hypervariable regions 3 to 5 (V3 to V5) diversity was directly associated with duration of patient survival, positive selection for change, and inversely correlated with the rate of disease progression as measured by the slope of CD4+ T cell loss [9]. Another study that examined Env C2-V5 sequences in men followed for 6 to 12 years following seroconversion demonstrated a complex pattern of viral diversity characterized by an early phase of linear increases in divergence and diversity, followed by an intermediate phase with increase in divergence but stabilization or decline of diversity, and a final phase showing stabilization or reduction in divergence and continued stability or decline in diversity [10]. In another study, analysis of C2-V5 Env sequences among typical progressors versus slow progressors showed that the typical progressors exhibited higher diversity, lower intra- and inter-sample divergence, evidence of lower host selective pressure and increases in both synonymous and non-synonymous substitutions over time while only non-synonymous substitutions increased in slow progressors [11].

The aforementioned studies and a comprehensive body of similar studies on HIV-1 diversity, divergence, and host selective forces that may impact on disease progression have been performed on HIV-1 subtype B [10,12-18]. Furthermore, these studies clearly demonstrate that patterns of Env diversity, divergence, and associated selective pressures identified can differ according to the stage of disease, the sampling methodology, the region of Env analyzed, the founder virus, and the host genetic background.

HIV-1 subtype C is the most rapidly spreading subtype worldwide [19,20], and an effective global vaccine will have to show efficacy against this subtype. A number of studies have explored Env diversity and diversification within HIV-1 subtype C [21,22] but data on this subtype remain relatively limited, despite accumulating evidence that this subtype may differ significantly from HIV-1 subtype B in certain biological properties mediated by the Env gene [21-25]. In particular, possible differences in Env diversity, divergence, and selective pressures between HIV-1 subtype C-infected individuals with divergent rates of disease progression remain understudied.

In this study, we used single genome amplification and sequencing to explore the evolution of the Env gp160 protein. Specifically, we investigated differences in diversity and divergence in 4 slow progressors and 4 progressors of black African descent infected with HIV-1 subtype C. Further, we investigated differences in Env features such as the extent of putative N-linked glycosylation, lengths of the variable and constant regions of gp160, and positive selection in slow-progressors and progressors in order to assess the correlation of these variables with rates of disease progression.

## Materials and methods

### Participants

Participant samples were retrospectively identified from the Sinikithemba cohort, which is a prospective natural history study of HIV-1 infected individuals based at McCord Hospital, Durban, South Africa as previously reported [26]. Ethics approval was obtained from the University of KwaZulu-Natal Biomedical Research Ethics Committee and all participants gave written informed consent to participate in the study. CD4 counts were performed at three month intervals whereas viral loads were done at six month intervals.

For this substudy, CD4 count was chosen as the primary determinant of disease progression for stratification into slow progressor and progressor categories. Both slow progressors and progressors were selected on the basis of a CD4 cell counts >500 cells/μl at study entry time point. However, at study exit, slow progressors maintained a CD4 count above 500 cells/μl or a viral load less than 10,000 viral RNA copies/ml. In contrast, progressors declined in CD4 counts to below 500 cells/μl and had a viral load above 10,000 copies/ml. The overall average follow up time was 19.5 months. All individuals were antiretroviral therapy naive before and during the window of evaluation. When the virological and immunological data became available beyond the study window (follow-up of an average of 39.8 months for slow progressors and 36.8 months for progressors, we analyzed these parameters relative to the study entry criteria and they remain statistically different for the progressors only (p = 0.03 for both CD4 and viral load).

#### Sample Collection, CD4 T cell counts and Plasma Viral Load

Blood was drawn from each subject into EDTA tubes and plasma was separated by centrifugation and stored at −80°C until use. Viral load was measured using the Amplicor Version 1.5 assay (Roche, Alameda CA, USA). CD4+ T-cell counts were enumerated by Trucount technology on a four colour FACS Calibur flow cytometer (Becton Dickinson, Franklin Lakes, New Jersey, USA).

### cDNA synthesis and single genome amplification

HIV-1 RNA extraction, cDNA synthesis, and single genome amplification were performed as previously reported with some modifications[27]. Briefly, primers were designed for the efficient amplification of HIV-1 subtype C envelope through nested PCR. For the first round PCR, the external primers used were VIF1: 5'-GGGTTTATTACAGGGACAGCAGAG-3' (HXB2 positions 4900-4923) and OFM19: 5'-GCACTCAAGGCAAGCTTTATTGAGGCTTA-3' (HXB2 positions 9604-9632). Primers for the second round PCR reaction were ENV A: 5'-GCTTAGGCATCTCCTATGGCAGGAAGAA-3' (HXB2 positions 5954-5982) and ENV N: 5'-CTGCCAATCAGGGAAGTAGCCTTGTGT-3' (HXB2 positions 9145-9171) [27]. Cycling conditions for first round PCR were as follows: 94°C for 4 min, 35 cycles of 94°C for 15 sec, 55°C for 30 sec, 68°C 4 min, and final extension of 68°C for 20 min followed by hold at 4°C. Second round PCR conditions were as follows: 94°C for 2 min, 45 cycles of 94°C for 15 sec, 55°C for 30 sec, 68°C for 4 min; final extension at 68°C for 20 min and 4°C hold. PCR products were visualized on a 1% agarose gel and amplicons were purified using the QIAquick PCR Purification Kit (Qiagen).

### Sequencing analysis of gp160

The full-length envelopes were sequenced in the forward and reverse directions using the ABI Prism Big Dye Terminator Version 3.1 cycle sequencing kit (Applied Biosystems, Foster City, CA), utilizing primers spanning the entire envelope and approximately 300 bp apart. Sequences were then resolved on the ABI 3130 XL genetic analyzer. Contigs were assembled and edited using the Sequencher v 4.8 software (Genecodes, Ann Arbor, MI). The sequences were aligned using Clustal W [28] and manually edited in the Genetic Data Environment (GDE 2.2). For phylogenetic analysis, subtype reference strains were obtained from the Los Alamos HIV sequence database http://www.hiv.lanl.gov/content/sequence/NEWALIGN/align.html). Phylogenetic trees were generated in PAUP*4.0b10 using the TVM I + G model of substitution as determined by MODELTEST 3.7 [29]. Trees were rooted with a homologous region of Group O reference (O.CM.96). Maximum likelihood (ML) trees of sequences from individual patients were also drawn using the appropriate evolutionary model (as determined by MODELTEST 3.7) and rooted with the "Best-fit root" as determined by Path-O-Gen v1.2 [30]. All trees were bootstrapped with 1,000 sampling replicates. Trees were viewed with FigTree v1.1.2 [30]. The approximate time of HIV-1 infection was estimated using BEAST (Bayesian Evolutionary Analysis Sampling Trees) version 1.4.8 (http://beast.bio.ed.ac.uk) in order to predict approximate time of infection prior to study enrollment [31]. BEAUTi was used to generate the .xml file to generate the BEAST file. The GTR substitution model with estimated base frequencies and a site heterogeneity model of gamma + invariant sites were used. A relaxed, uncorrelated lognormal molecular clock model was chosen. The MCMC (Monte Carlo Markov Chain) length of chain was set at 30,000,000 to give an effective sample size (ESS) > 170. The number and location of putative N-linked glycosylation sites (PNGs) were estimated using N-GlycoSite (http://www.hiv.lanl.gov/content/sequence/GLYCOSITE/glycosite.html) from the Los Alamos National Laboratory database. Sequence diversity was calculated using the Maximum Composite Likelihood option in Mega 4.0 [32]. Characteristic differences between progressors and slow progressors including corresponding study entry and exit time-points were identified using VESPA (Viral Epidemiology Signature Pattern Analysis) [33]. Nucleotide substitution rates were calculated using baseml from the PAML software package [34]. Sites under positive selection were identified using the SLAC option in HyPhy [35] and CODEML as implemented in the PAML software package.

Positively selected sites and signature mutations were mapped onto the X-ray structure of a clade C HIV-1 gp120 (3LQA.pdb) [36] using the BIOPREDICTA module in the VLifeMDS software package (VLife Science Technologies, 2007). Gp41 was modeled in SWISS-MODEL [37] using 1ENV.pdb [38] as a template. Structures were rendered and annotated in PyMol [39].

### Statistical analyses

Pairwise comparisons of different parameters including genetic diversity, PNGs, and length polymorphism between subjects in the two groups were calculated by the Mann-Whitney non-parametric test using the GraphPad Prism 5 software programme unless otherwise stated. Correlations were regarded as statistically significant with a *p *value < 0.05. All reported *p *values are for two-sided tests.

### Genebank accession numbers

Sequences have been assigned the following GenBank accession numbers: GU216702-GU216737 and GU216739-GU216847.

## Results

### Study participant characteristics

**Figure 1:**
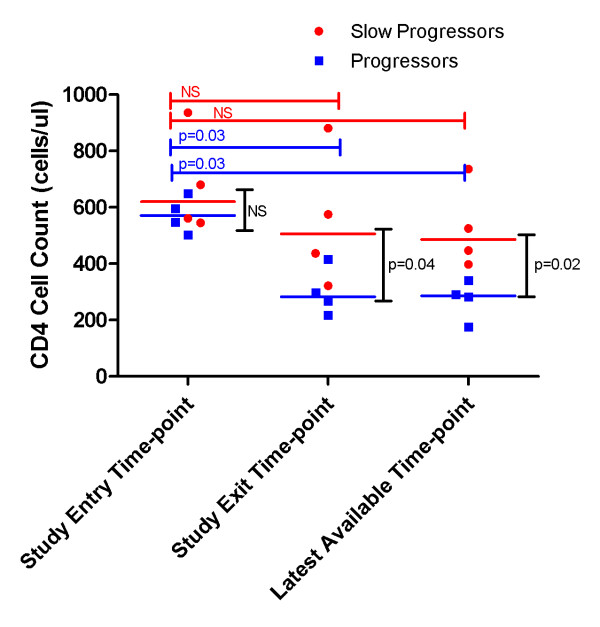
**CD4 of study entry, study exit and latest available time-point data for slow progressors and progressors**. The red circles depict the data points for the slow-progressors. The blue squares depict data points for the progressors. Red bars and blue bars represent the *p *values for the slow progressors and progressors respectively. Black bars represent *p *values for inter-group comparison for the different time-points. NS = not significant. All comparisons between the study entry, study exit and latest available time-point parameters were performed using the Mann-Whitney unpaired t test, and *p *values are shown. Differences were regarded as statistically significant with a *p *value < 0.05. When slow progressors were compared to progressors, the analysis yielded significant differences when the CD4 at study exit and last available time-points were compared - as shown above (p = 0.04 and p = 0.02 respectively). Likewise viral load was significantly different between the groups at study exit and the latest available time-point (p = 0.03 and p = 0.02 respectively, data not shown).

There were eight participants in this study, seven female and one male. The average age of the participants was 34 years old (range: 22-59 years). At study entry, both progressors and slow progressors did not differ in their CD4 T cell counts (medians of 621 cells/μl versus 571 cells/μl (p = 0.39) as shown in figure 1. However, at study exit the median CD4 count of slow progressors was 506 cells/μl, which is not significantly different from the CD4 count at study entry (p = 0.7), while the progressors' median CD4 count had significantly declined to 283 cells/μl, (p = 0.03). Slow progressors also had no significant difference for viral load (p = 1.0, data not shown) between study entry and exit time-points, whereas progressor participants had significantly lower viral load (p = 0.03, data not shown) at study entry compared to exit time-point. In addition, CD4 (figure 1) and viral load (data not shown) were statistically different for progressors only at the latest available time-point compared to study entry (p = 0.03 for both parameters). Furthermore, we used BEAST to estimate the approximate time of infection in both groups of participants. Slow progressors were estimated to be infected for a mean period of 8.2 years (range 4.75-15 years) compared with 2 years (range 0.75-3.75 years) for progressors.

### Phylogenetic relationships

**Figure 2:**
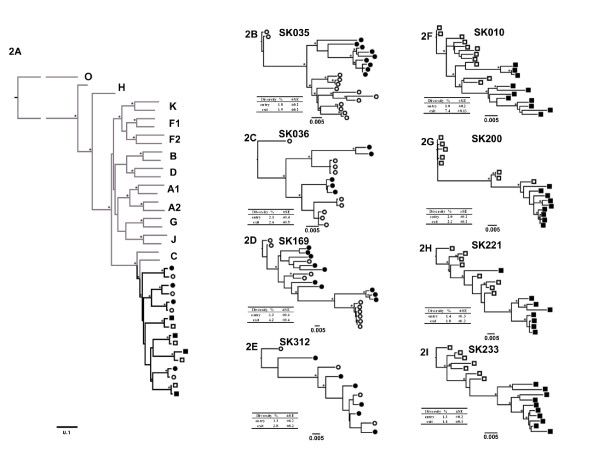
**Maximum Likelihood trees of SGA-derived full-length *env *sequences from Progressors and Slow progressors**. Figure 2A Subtype tree of consensus sequences for slow progressors entry (●) and exit (○) and progressors entry (■) and exit (□) time-points. Subtype reference strains were obtained from the Los Alamos database (http://www.hiv.lanl.gov/content/sequence/NEWALIGN/align.html). The tree was rooted with Group O as the outgroup. Figures 2B to 2E represent maximum likelihood trees for the slow progressor sequences and Figures 2F to 2I represent trees for the progressor sequences. All trees were drawn in Paup* using the appropriate substitution model. Bootstrap support from 1000 bootstrap resamplings is indicated by ●. Only values >70% are shown. The scale bar is shown at the bottom of figure 2A is 0.1 and for figures 2B-2I the scale bar is 0.005. The mean study entry and exit intra-patient nucleotide diversity and the standard error of (SE) for both the groups are shown in the tables below the individual trees.

To analyze phylogenetic relationships and changes in envelope sequences in slow progressors and progressors over a period 19.5 month follow-up, a mean of 9 single genome full-length gp160 amplicons per participant per timepoint(range 4-11 amplicons) for the study entry and exit time-point were analyzed, for a total of 146 sequences. One of the slow-progressors (SK312) had a few putative functional Env amplicons which were included in the final analysis when compared to the other study participants. This was due to a low number of SGA-derived clones which was limited by the low viral load and plasma sample availability. All participants' consensus sequences bootstrapped confidently with subtype C reference strains, as determined by a Maximum Likelihood tree for each patient at each time point (Figure 2A). As expected, consensus sequences from the study entry and study exit for each patient formed monophyletic groups.

Overall, there were no distinguishing phylogenetic patterns noted between sequences from the slow progressors and progressors (Figure 2A). Slow progressors showed a more diverse pattern characterized by either separate (sub)clusters at study entry and exit (Figure 2B - SK035) or intermingling of sequences from early and exit time points (Figure 2E - SK312). Additionally, phylogenetic clusters at study exit typically showed similar (Figure 2C - SK036) or longer branch length (Figure 2D, example subject - SK169), compared with that of the study entry sequences. However, individual participant sequence trees for the progressors tended to show segregation between entry and exit time-point sequences (Figures 2F-I).

### Intra-patient diversity analysis

Intra-patient diversity, defined as the mean pair-wise nucleotide distance, was calculated by measuring distances between all sequences from a single individual at a single time-point, and is shown alongside the phylogenetic trees (Figures 2B-I). Mean overall intra-patient diversity was 2.75% for the four slow progressors and 2.21% for the four progressors (p = 0.07). The mean baseline intra-patient nucleotide diversity for the slow progressors was 2.63% (range 1.8-3.3%) and 1.42% (range 1.0-2.0%) for the progressors, but this did not reach statistical significance (p = 0.08). Study exit time point mean intra-patient diversity was 2.88% (range 1.9-4.2%) and 3.0% (range 1.0-7.4%) for slow progressors and progressors, respectively, which was not a significant difference (p-value = 0.56). Collectively, these data show that in this cohort, slow progressors trended to higher intra-patient sequence diversity compared to progressors although the differences did not reach statistical significance.

### Nucleotide substitution rates in study entry and exit in slow progressors and progressors

To examine the evolution of the envelope gene over the study period, we calculated the rate of nucleotide divergence for each patient's *env *sequences. On average the nucleotide substitution rate was higher in the progressors (1.2 ×10^-2 ^nucleotide substitutions/site/year; range 6-17 ×10^-3^), compared to the slow progressors (3 ×10^-3 ^nucleotide substitutions/site/year; range 0.1-7 ×10^-3^), but did not differ significantly (p = 0.12). The nucleotide substitution rate appeared to follow the viral load pattern, such that there was a positive but non-significant linear correlation between divergence (nucleotide substitution rate) and the log_10 _viral load (p = 0.12) - data not shown.

### Heterogeneity of diversity in Env in slow progressors and progressors for the variable and constant regions

**Figure 3:**
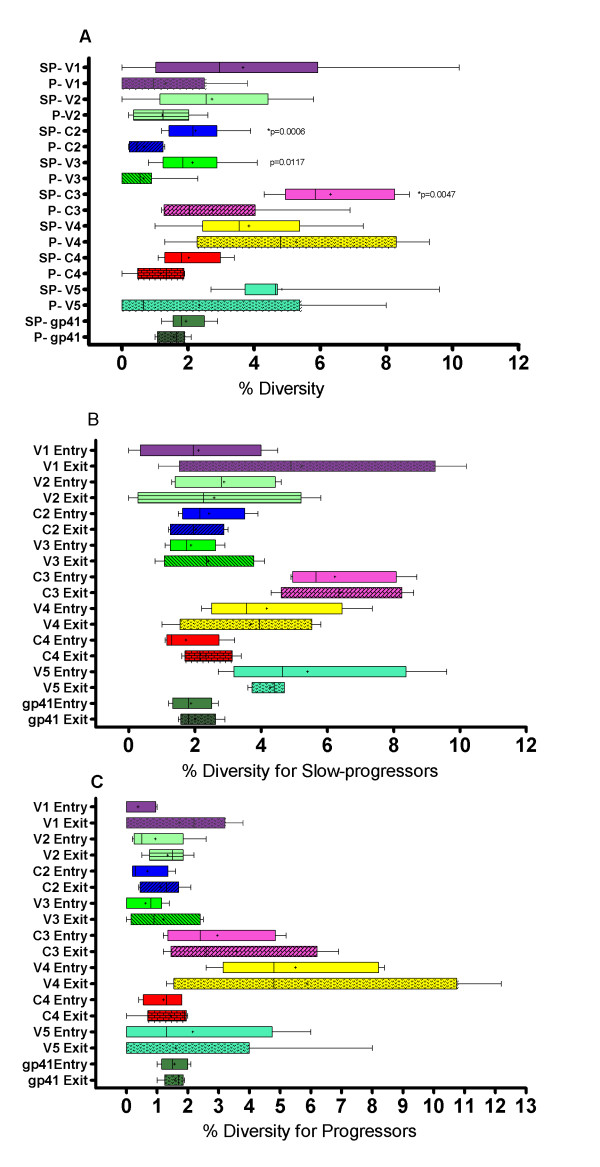
**Box-and-whisker plots of genetic diversity of the dissected envelope gene for V1, V2, C2, V3, C3, V4, C4 and V5 and gp41 for slow progressors and progressors**. The whiskers extend to the upper and lower adjacent values. Comparisons between the groups were done with the Mann Whitney unpaired t test, and *p values *are shown. Correlations were regarded as statistically significant with a *p *value < 0.05 and only significant *p *values are shown. *p *values depicted with an asterisk (*) indicate the ones corrected for multiple comparisons using the Bonferroni correction of p ≤ 0.006. Mean diversity value is depicted as (+). Figure 3A Diversity of V1, V2, C2, V3, C3, V4, C4, V5 and gp41 in slow progressors (SP) and progressors (P) overall. Figure 3B Box and whisker plots of intra-patient diversity analysis for slow progressors for different regions of the Env gene for study entry and study exit. Figure 3C Box and whisker plots of intra-patient diversity analysis for progressors for different regions of the Env gene for study entry and study exit.

To assess whether there were overall differences in diversity between regions of *env *at study entry and exit, we analyzed distinct regions of the *env *gene separately and compared diversity scores between the slow progressors and progressors for the five variable loops, three constant regions and gp41 over time as seen in Figure 3A. Significant diversity differences between slow progressors and progressors were noted for the C2 (p = 0.004), V3 (p = 0.01) and C3 (p = 0.005), with differences remaining significant for C2 and C3 even after applying Bonferroni correction for multiple comparisons (≤ 0.006). There was no significant difference in overall inter-patient percentage diversity between slow progressors and progressors for V1 (p = 0.12), V2 (p = 0.09), V4 (p = 0.29), C4 (p = 0.13), V5 (p = 0.08) and gp41 (p = 0.40).

Next, we assessed the differences in inter-individual *env *diversity patterns across *env *for study entry and exit time-points. The results of this analysis are summarized in Figure 3B for slow progressors and Figure 3C for progressors. There were no significant differences between the early and exit time-point intra-patient diversity for either of the groups in any of the regions.

### Length polymorphisms and glycosylation patterns for the variable and constant regions

Overall length of certain regions and changes in the number of N-linked glycosylation sites (PNGs) in Env have been shown to influence the sensitivity or resistance of the virus to antibody neutralization and may also influence efficiency of interactions with receptors on the cell surface [7,40]. However, these characteristics have not been comprehensively analyzed for HIV-1 subtype C and most studies have focused on the V3 loop, which is an important but not exclusive determinant of viral tropism and cell entry [41]. We sought to determine whether Env sequence characteristics are associated with disease progression in HIV-1 subtype C. Table 1 depicts Env region length polymorphisms and numbers of PNGs in slow progressors and progressors over time. Mean V1-V2 length for progressors and slow progressors was 66 amino acids and 69 amino acids respectively (Table 1) but this difference was not statistically significant (p = 0.32). Similarly, we observed no differences in C4-V5 amino acid length (p = 0.29) or PNGs (p = 0.15), and length polymorphism for C2-V3 showed no significant difference between the groups. However, a significant difference was noted in the overall number of PNGs in C2-V3 between slow progressors and progressors (p = 0.009), a result that remained significant after Bonferroni test correction (p < 0.01). For C3-V4, slow progressors had a significantly higher mean of 85 (range 81-90) compared to 82 (range: 76-88) amino acids in progressors (p = 0.02), however analysis of PNGs indicated no difference between the groups (p = 0.96). Interestingly, there was a significant difference overall between the groups in the numbers of PNGs for C3 only in the progressors compared to the slow progressors (p = 0.0006) (data not shown). V1-V4 length overall was significantly different, with slow progressors displaying longer V1-V4 length of 286 amino acids (range 282-294) compared to progressors' 281 (range 276-292; p = 0.009). In contrast, we found that the numbers of PNGs for V1-V4 overall was significantly higher with a mean of 22, (range 20-23) in progressors compared to a mean of 20 (range 19-21) in slow progressors (p = 0.02). Gp41 length was significantly higher in progressors (range 245-252) compared to slow progressors (range 239-252; p = 0.02) (Table 1). However, the number of PNGs in gp41 in slow progressors (range 3-5) was statistically different from those of progressors (range 2-4 PNGs; p = 0.02).

**Table 1:** Env sequence characteristics of amino acid length and potential N-linked glycosylation sites for slow progressors and progressors^#^

*Patient*	*V1V2*		*C2V3*		*C3V4*		*C4V5*		*gp41*	
	mean length (range)	mean PNGs (range)	mean length (range)	mean PNGs (range)	mean length (range)	mean PNGs (range)	mean length (range)	mean PNGs (range)	mean length (range)	mean PNGs (range)
**Slow progressors**										
**SK035 entry**	69 (62-72)	6 (3-7)	133	8	80 (75-81)	7 (5-8)	53 (52-56)	3 (3-4)	252	5 (3-5)
**SK035 exit**	69 (59-70)	6 (4-8)	133	8	82 (80-88)	7 (6-8)	53 (52-58)	3 (2-4)	250(243-252)	5 (4-5)
**SK036 entry**	64 (61-73)	5 (4-6)	133	6 (7-8)	84 (82-84)	8 (8-9)	52	3 (2-3)	243(243)	4 (3-5)
**SK036 exit**	66 (59-73)	4 (3-6)	133	8 (7-8)	84	8 (7-9)	52	3 (2-3)	243(243)	5 (4-5)
**SK169 entry**	75 (71-80)	6 (5-7)	133(132-133)	6 (6-8)	85 (84-88)	7 (6-8)	54 (52-55)	3 (2-4)	245(241-245)	3 (3-4)
**SK169 exit**	76 (71-77)	7 (6-7)	133	6 (6-8)	86 (84-95)	7 (4-10)	54 (51-55)	3 (2-4)	245(245)	3 (3-4)
**SK312 entry**	66 (60-69)	5(3-5)	133	6	90 (85-97)	9 (5-11)	51 (50-54)	3 (2-4)	239(233-252)	3
**SK312 exit**	67 (67-69)	5	133	6	90 (84-97)	8 (4-10)	51 (50-55)	3 (1-4)	239(236-252)	3
Mean (range) over time	69 (64-75)	6 (4-7)	133	7 (6-8)	85 (81-90)	8 (7-9)	53 (51-54)	3	245(239-252)	4 (3-5)
**Progressors**										
										
**SK010 entry**	65	6	133	8	79 (77-82)	8 (7-9)	52 (52-53)	3	252	3
**SK010 exit**	65 (65-66)	6	133	8	78 (75-79)	7 (5-8)	52 (50-54)	3	252	3
**SK200 entry**	66 (64-78)	6 (6-7)	133	8	76 (75-76)	6 (6-7)	52	2 (2-3)	252	3 (2-3)
**SK200 exit**	73 (71-73)	6 (6-8)	133	8	76 (75-76)	7	52	3	252	3 (2-3)
**SK221 entry**	72 (55-74)	7 (3-8)	133	9	77 (73-82)	7 (7-8)	51	3 (3-4)	252	2
**SK221 exit**	71 (63-76)	5 (4-5)	133	9	85 (74-90)	8 (6-9)	51	3 (3-4)	246(245-252)	2
**SK233 entry**	58	4	133	8	84 (84)	9 (8-9)	52 (50-51)	3	245	3 (3-4)
**SK233 exit**	59 (59-63)	5 (5-6)	133	8 (7-8)	84 (84)	9 (8-9)	53 (52-53)	3 (2-4)	245	3 (3-4)
Mean (range) over time	66 (59-72)	6 (4-7)	133	8 (8-9)	82 (76-88)	8 (7-9)	52 (51-53)	3 (2-4)	250(245-252)	3 (2-3)
*p Value*	***p = 0.32***	***p = 0.78***	***NS***	*p = 0.009	***p = 0.02***	*p = 0.96*	***p = 0.29***	***p = 0.15***	***p = 0.02***	***p = 0.02***

***p *value **was calculated using the two-tailed Mann-Whitney non-parametric test overall between the slow progressors and progressors.

Where only the mean is reflected it is because it is equivalent to the range.

* represents the *p *value that remained significant after Bonferroni adjustment for multiple comparisons (p < 0.01), NS represents a non-significant *p *value. Potential N-linked glycosylation = PNGs.

**^# ^Data for V1-V4 length is as follows: slow progressors had a mean of **286 amino acids (range 282-294) versus progressors' 281 amino acids (range 276-292; p = 0.009).

**^# ^Data for V1-V4 PNGs is as follows: slow progressors had a mean of **20 **PNGS **(range 19-21) versus a mean of 22 PNGs (range 20-23) in progressors (p = 0.02).

### Positive selection pressure

The dN/dS (ω) ratio reflects non-synonymous (dN) substitutions to synonymous (dS) substitutions per codon site, with a value of >1 at any site indicating positive selection pressure [42]. The ω values for the whole of gp160, as well as the variable and constant regions within envelope, were calculated using the M1a and M2a models implemented in CODEML. The settings for the M1a (neutral) model were: model = 0, NSsites = 1, and for the M2a (selection) model were: model = 0, NSsites = 2. A Likelihood Ratio Test (2ΔlnL) was performed between the likelihood scores of the M1a (null) vs. M2a (alternative) models. A χ^2 ^test was performed using two degrees of freedom [34]. For V1, the M2a (selection) model was supported only in the slow progressors (p < 0.005). For V2 and V3, the null hypothesis (M1a) could not be rejected for both slow and typical progressors (p = 0.25), while the M2a model was supported for all remaining envelope regions (p < 0.005) for both groups.

**Figure 4:**
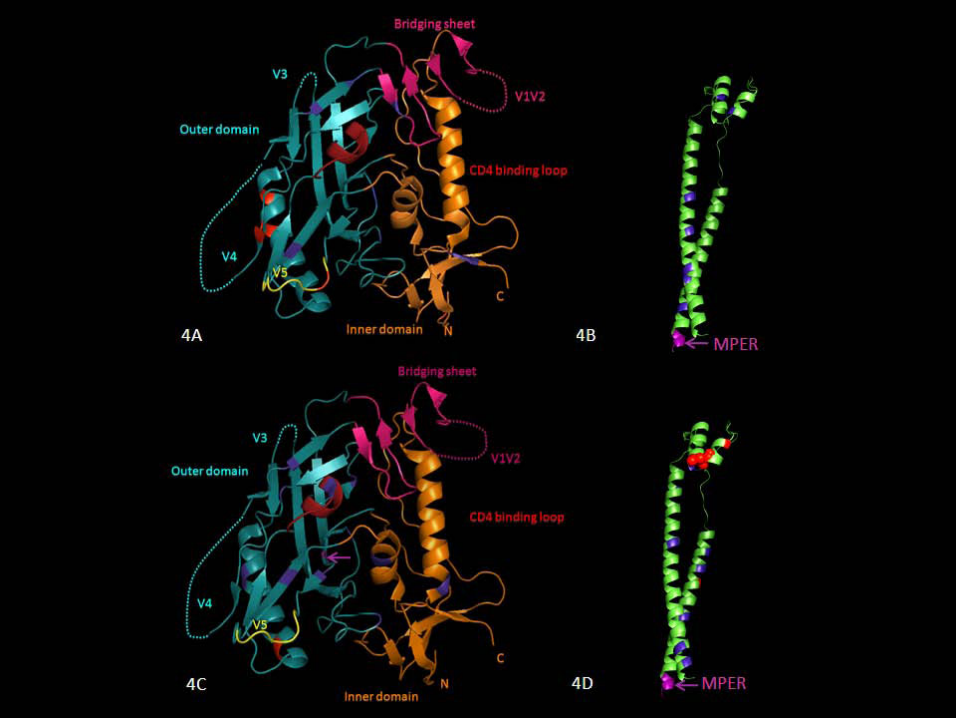
**Three dimensional structural illustrations of positions associated with positive negative and neutral selection**. Locations were mapped onto a model of gp120 based on the X-ray structure of the gp120 core in complex with sCD4 and 21c Fab (3LQA.pdb) for slow progressors - Figure 4A and for progressors - Figure 4C. V1V2 and V3 loops were drawn onto the core for completeness. In the orientation shown, the cellular and viral membranes would be located above and below the protein respectively. Figure 4B and 4D represent ribbon structures of gp41 for slow progressors and progressors with the MPER region highlighted. Cartoon diagrams showing locations under positive selection, as determined by dN/dS ratios for subtype C sequences. Red indicates strong positive selection (dN/dS >4) as shown above in HXB2 positions 87, 336, 340, 396, 410 and 460 for slow progressors (Figure 4A) and in progressors at positions 350 (Figure 4C) and 607, 612 and 641 in Figure 4D. Blue indicates strongly negatively selected positions (<-3). Purple and purple arrows denote changes in putative functional sites as shown in Figures 4B, 4C and 4D. Spheres indicate signature sequence differences. It should be noted that the gp120 core crystal structures which were modeled on the 3LQA.PDB structure, include amino acid residues from HXB2 position 86-491. The gp41 structure based on 1ENV.pdb includes amino acid residues from HXB2 position 541-662. Therefore all the positively and negatively selected sites are not indicated on the gp120 and gp41 structures.

Analysis of the entire Env gp160 in the two groups using CODEML and the SLAC option in HYPHY identified 9 common sites under positive selection in slow progressors and 5 sites in progressors. In slow progressors (Figures 4A and 4B), these were at codons 87, 138 and 140 (V1), 336 and 340 (C3), 396 and 410 (V4), 460 (V5) and 832 (gp41). Most of the sites under positive selection in slow progressors were either adjacent to a putative N-linked glycosylation site (codons 87, 138, 336 and 410) or were located at N-linked glycosylation sites (codons 140, 340, 396 and 460). Interestingly, positions 336 and 340 are within the α-2-helix (HXB2 position 335-352); it has been previously reported that changes within this region may confer autologous antibody neutralization resistance [19].

For progressors (Figures 4C and 4D), 4 of 5 positively selected sites were located in gp41 (codons 607, 612, 641 and 821), while the remaining site, codon 350, was located in the α-2-helix of C3 immediately downstream of V3. Two of the sites under positive selection in the progressors were either adjacent to, (codon 612) or located at a putative N-linked glycosylation site (codon 641).

One additional site identified using CODEML, codon 671, is located at a linear epitope NWFNIT, which is within the membrane proximal external region (MPER) of gp41, an epitope that is well recognized by a broadly neutralizing antibody (4E10) [43].

### Signature sequence differences between slow progressors and progressors

To identify key differences between the groups, consensus sequences of slow progressors and progressors study entry and exit were generated in VESPA using an 80% threshold (i.e. sequence differences were in >80% of the sequences). Signature differences were noted at 6 amino acid positions between the progressors and slow progressors consensus sequences. Four of six of these differences occurred in gp41 (codons 607, 727, 770 and 837), and the remaining two were at codons 80 and 133. No signature differences were noted between the entry and exit time points within each group.

Except for an N to S/D mutation in the progressors at codon 80, which resulted in the gain of a casein-kinase-2 (CK2) phosphorylation site at codons 77-80, most of the signature changes were not at putative functional sites. Other changes, although not in the signature, but resulting in a change in putative functional sites in the progressors, are: a V to T mutation at codon 455 resulting in the gain of a myristoylation site at codon 451-456, a Q to K mutation at codon 665 (within the ALDSQWN epitope) resulting in the gain of a tyrosine kinase phosphorylation (TKP) site at codons 665-667, and an N to S mutation at codon 671 resulting in the gain of a CK2 phosphorylation site at codons 671-674 within the NWFDIT epitope. Interestingly, the loss of a putative N-linked glycosylation site in the progressors in the V4 region was compensated for by a gain of an N-linked glycosylation site in the C3 region (codons 362-365). When these signature patterns were compared with the subtype B reference strain, it was noted that an L to V mutation at codon 800 in the subtype C signature sequences resulted in a loss of a putative leucine zipper (codons 793-814). Whether the gain or loss of putative functional sites influence viral pathogenesis needs to be confirmed with functional assays.

## Discussion

In this study we aimed to identify *env *sequence characteristics that may distinguish progressors from slow progressors in a chronically HIV infected anti-retroviral naïve subtype C-infected cohort. We used a single genome amplification approach in order to accurately and comprehensively represent the diversity of viral quasi-species. Several indicators of evolutionary forces were used to elucidate putative differences between the groups including heterogeneity of envelope sequence diversity, Env length polymorphisms, numbers of PNGs, positive selection, and signature sequence characteristics.

Our study suggests that regions of Env are shaped by different evolutionary forces which may in turn leave viral sequence footprints that may distinguish slow progressors from progressors in chronic HIV-1 subtype C infection. It has previously been shown that in subtype B infection there may be Env region-specific differences in evolutionary forces between those with high versus low viral loads [9]. Our study demonstrated a non-significant trend towards increased intra-patient diversity in slow progressors, a finding consistent with other studies on HIV disease progression [44-46]. In contrast, a study of primary HIV-1 subtype C infection has found that increased envelope diversity is inversely correlated with CD4 T cell counts and is associated with rapid disease progression [47]. Together, these results may imply that evolutionary forces that drive HIV-1 subtype C diversification differ according to the phase of infection. On close examination of the envelope regions we found that diversity in C2, V3 and C3 was higher in slow progressors compared to progressors suggesting co-evolution of these regions. These findings are consistent with findings from other studies [48,49]. From a functionality standpoint it appears that, because the V3 loop is very important for viral entry, increased diversity in this region is a correlate of viral attenuation [24].

Length polymorphisms in the constant and variable envelope regions may also contribute to structural diversity in terms of glycan packing and protein folding of the virion structure. An unusual finding was that the longer V1-V4 in slow progressors had fewer PNG's whereas the longer gp41 domain contained fewer PNGs in progressors. Several studies have shown the association between neutralization sensitivity and shorter V1-V4 length [50,51]. In contrast, other studies have shown longer V1-V4 with extensive glycosylation mask neutralizing antibody sensitive epitopes in subtype C [6]; however, in subtype B no such association was found [52]. Our observations may imply that longer length regions may be masking neutralization sensitive epitopes as suggested by Gray et al. [47]. Additionally in progressors, a loss of a glycan in V4 was compensated for by a gain in a PNG within C3, implying a shifting glycan shield as suggested previously [7].

High dN/dS ratios indicative of strong diversifying selection due to humoral immune pressure [42], occurred mainly within gp41 in progressors, while slow progressors had a number of regions targeted. This suggests that the nature of antibody targets may differ between the groups. Interestingly, both groups had positive selection in the α-2-helix within C3. It has been suggested that, because the V4 loop is shorter in subtype C than in subtype B, the α-2 helix is more exposed and more antigenic [49,53,54]. Interestingly, position 607 of gp41 was positively selected in progressors and was also a signature sequence difference between progressors and slow-progressors, indicating that there may be putative humoral immune pressure driving escape at that position. Additionally, gp41 in progressors showed differences at two putative antibody sites. Firstly, ELDKWAS was recognized by neutralizing antibody (nAb) 2F5, where DKW are the sentinel amino acids that determine sensitivity to 2F5 [43]. This appears in the majority of the slow progressors's sequences; however, it is substituted by DSW in all the progressors indicating a loss of a putative antibody recognition site. In addition there is a sequence change from Q at position 665 to K, making the overall progressor sequence ALDSWKN. Secondly, an N to S change at codon 671, which is within a linear epitope- **N**WFNIT- that is recognized by nAb 4E10, may result in a loss of this recognition site. In addition, this codon was positively selected for in the progressors. The effect of the loss of these putative recognition sites during chronic disease progression is unknown. We propose that the high antigenic stimulation in progressors may elicit antibodies whose antiviral effectiveness may be limited. Together these results may imply that the virus uses multiple strategies to evade the immune system, including increased V1-V4 amino acid length, increased numbers of PNGs, and specific mutations resulting in the virus gaining selective advantages. Essentially, the cat and mouse game that persists during chronic infection as a result of the dichotomy between antigenic stimulation and immunological response, which impacts and influences viral characteristics, needs further investigation.

The limitations of the study are that firstly, we do not know the exact time of infection for these subjects. Therefore stratification of study subjects as progressors or slow progressors relied on short-term (19.5 months) follow-up immunological data, which may be an unrepresentative snap-shot of the entire natural history of disease progression for these participants. However, this concern was somewhat allayed by bioinformatic analysis of the study sequences that showed that consistent with the stratification, progressors in this cohort were more likely to have been infected for shorter period of time than slow progressors. Second, the sample size of the study cohort was relatively small, which may have limited our statistical power to identify differences. Third, we had a limited number of SGA-generated amplicons for one of the study participants in particular, due to their low viral load and sample volume limitation. In addition, many more *env *amplicons were generated than were included in the final analyses as some of the amplicons had sequences with stop codons. Fourth, although the slow progressors and progressors differed in markers of disease progression at study exit, more stringent selection criteria could potentially identify additional significant differences. Overall, therefore, the findings reported here will require duplication in larger cohorts with longer periods of follow-up and more significant differences in immunological and virological outcomes.

## Conclusions

The dynamics of HIV-1 *env *evolution between chronic slow progressors and progressors are distinct. Single genome sequence analysis of circulating viruses in slow progressors and progressors indicate that diversity, Env length polymorphisms, sites under positive selection pressure, and PNGs consistently map to specific regions in slow progressors or progressors. Varied diversity across the *env *genome, the relationship between amino acid length, number of PNGs or sites under positive selection may provide further insight to the intrinsic differences between the viruses from both groups and the influence of the host's selective pressures which may be used to inform more effective vaccine design.

## Competing interests

The authors declare that they have no competing interests.

## Authors' contributions

DA and TN conceived the experiments. DA, MG and TG carried out the experiments. DA, MG and TN wrote the paper and all authors helped interpret the data and reviewed the manuscript.

## References

[B1] Mansky LM Temin HM Lower in vivo mutation rate of human immunodeficiency virus type 1 than that predicted from the fidelity of purified reverse transcriptase J Virol 1995 69 5087 5094 7541846 10.1128/jvi.69.8.5087-5094.1995 PMC189326

[B2] Preston BD Poiesz BJ Loeb LA Fidelity of HIV-1 reverse transcriptase Science 1988 242 1168 1171 10.1126/science.2460924 2460924

[B3] Brander C Frahm N Walker BD The challenges of host and viral diversity in HIV vaccine design Curr Opin Immunol 2006 18 430 437 10.1016/j.coi.2006.05.012 16777397

[B4] Walker BD Burton DR Toward an AIDS vaccine Science 2008 320 760 764 10.1126/science.1152622 18467582

[B5] Resch W Hoffman N Swanstrom R Improved success of phenotype prediction of the human immunodeficiency virus type 1 from envelope variable loop 3 sequence using neural networks Virology 2001 288 51 62 10.1006/viro.2001.1087 11543657

[B6] Rademeyer C Moore PL Taylor N Martin DP Choge IA Gray ES Sheppard HW Gray C Morris L Williamson C Genetic characteristics of HIV-1 subtype C envelopes inducing cross-neutralizing antibodies Virology 2007 368 172 181 10.1016/j.virol.2007.06.013 17632196

[B7] Wei X Decker JM Wang S Hui H Kappes JC Wu X Salazar-Gonzalez JF Salazar MG Kilby JM Saag MS Antibody neutralization and escape by HIV-1 Nature 2003 422 307 312 10.1038/nature01470 12646921

[B8] Nowak MA Anderson RM McLean AR Wolfs TF Goudsmit J May RM Antigenic diversity thresholds and the development of AIDS Science 1991 254 963 969 10.1126/science.1683006 1683006

[B9] Wolinsky SM Kunstman KJ Safrit JT Koup RA Neumann AU Korber BT Response: HIV-1 Evolution and Disease Progression Science 1996 274 1010 1011 10.1126/science.274.5289.1010 17798610

[B10] Shankarappa R Margolick JB Gange SJ Rodrigo AG Upchurch D Farzadegan H Gupta P Rinaldo CR Learn GH He X Consistent viral evolutionary changes associated with the progression of human immunodeficiency virus type 1 infection J Virol 1999 73 10489 10502 10559367 10.1128/jvi.73.12.10489-10502.1999 PMC113104

[B11] Bagnarelli P Mazzola F Menzo S Montroni M Butini L Clementi M Host-specific modulation of the selective constraints driving human immunodeficiency virus type 1 env gene evolution J Virol 1999 73 3764 3777 10196271 10.1128/jvi.73.5.3764-3777.1999 PMC104154

[B12] Freel SA Fiscus SA Pilcher CD Menezes P Giner J Patrick E Lennox JL Hicks CB Eron JJ Jr Shugars DC Envelope diversity, coreceptor usage and syncytium-inducing phenotype of HIV-1 variants in saliva and blood during primary infection Aids 2003 17 2025 2033 10.1097/00002030-200309260-00003 14502005

[B13] Delwart E Magierowska M Royz M Foley B Peddada L Smith R Heldebrant C Conrad A Busch M Homogeneous quasispecies in 16 out of 17 individuals during very early HIV-1 primary infection Aids 2002 16 189 195 10.1097/00002030-200201250-00007 11807302

[B14] Gottlieb GS Heath L Nickle DC Wong KG Leach SE Jacobs B Gezahegne S van 't Wout AB Jacobson LP Margolick JB Mullins JI HIV-1 variation before seroconversion in men who have sex with men: analysis of acute/early HIV infection in the multicenter AIDS cohort study J Infect Dis 2008 197 1011 1015 10.1086/529206 18419538 PMC3419593

[B15] Keele BF Giorgi EE Salazar-Gonzalez JF Decker JM Pham KT Salazar MG Sun C Grayson T Wang S Li H Identification and characterization of transmitted and early founder virus envelopes in primary HIV-1 infection Proc Natl Acad Sci USA 2008 105 7552 7557 10.1073/pnas.0802203105 18490657 PMC2387184

[B16] Learn GH Muthui D Brodie SJ Zhu T Diem K Mullins JI Corey L Virus population homogenization following acute human immunodeficiency virus type 1 infection J Virol 2002 76 11953 11959 10.1128/JVI.76.23.11953-11959.2002 12414937 PMC136917

[B17] Ritola K Pilcher CD Fiscus SA Hoffman NG Nelson JA Kitrinos KM Hicks CB Eron JJ Jr Swanstrom R Multiple V1/V2 env variants are frequently present during primary infection with human immunodeficiency virus type 1 J Virol 2004 78 11208 11218 10.1128/JVI.78.20.11208-11218.2004 15452240 PMC521858

[B18] Frost SD Liu Y Pond SL Chappey C Wrin T Petropoulos CJ Little SJ Richman DD Characterization of human immunodeficiency virus type 1 (HIV-1) envelope variation and neutralizing antibody responses during transmission of HIV-1 subtype B J Virol 2005 79 6523 6527 10.1128/JVI.79.10.6523-6527.2005 15858036 PMC1091710

[B19] Rong R Gnanakaran S Decker JM Bibollet-Ruche F Taylor J Sfakianos JN Mokili JL Muldoon M Mulenga J Allen S Unique mutational patterns in the envelope alpha 2 amphipathic helix and acquisition of length in gp120 hypervariable domains are associated with resistance to autologous neutralization of subtype C human immunodeficiency virus type 1 J Virol 2007 81 5658 5668 10.1128/JVI.00257-07 17360739 PMC1900276

[B20] Coetzer M Cilliers T Papathanasopoulos M Ramjee G Karim SA Williamson C Morris L Longitudinal analysis of HIV type 1 subtype C envelope sequences from South Africa AIDS Res Hum Retroviruses 2007 23 316 321 10.1089/aid.2006.0207 17331039

[B21] Ping LH Nelson JA Hoffman IF Schock J Lamers SL Goodman M Vernazza P Kazembe P Maida M Zimba D Characterization of V3 sequence heterogeneity in subtype C human immunodeficiency virus type 1 isolates from Malawi: underrepresentation of X4 variants J Virol 1999 73 6271 6281 10400718 10.1128/jvi.73.8.6271-6281.1999 PMC112705

[B22] Tscherning C Alaeus A Fredriksson R Bjorndal A Deng H Littman DR Fenyo EM Albert J Differences in chemokine coreceptor usage between genetic subtypes of HIV-1 Virology 1998 241 181 188 10.1006/viro.1997.8980 9499793

[B23] Ball SC Abraha A Collins KR Marozsan AJ Baird H Quinones-Mateu ME Penn-Nicholson A Murray M Richard N Lobritz M Comparing the ex vivo fitness of CCR5-tropic human immunodeficiency virus type 1 isolates of subtypes B and C J Virol 2003 77 1021 1038 10.1128/JVI.77.2.1021-1038.2003 12502818 PMC140829

[B24] Abraha A Nankya IL Gibson R Demers K Tebit DM Johnston E Katzenstein D Siddiqui A Herrera C Fischetti L CCR5- and CXCR4-tropic subtype C human immunodeficiency virus type 1 isolates have a lower level of pathogenic fitness than other dominant group M subtypes: implications for the epidemic J Virol 2009 83 5592 5605 10.1128/JVI.02051-08 19297481 PMC2681953

[B25] Rong R Li B Lynch RM Haaland RE Murphy MK Mulenga J Allen SA Pinter A Shaw GM Hunter E Escape from autologous neutralizing antibodies in acute/early subtype C HIV-1 infection requires multiple pathways PLoS Pathog 2009 5 e1000594 10.1371/journal.ppat.1000594 19763269 PMC2741593

[B26] Kiepiela P Leslie AJ Honeyborne I Ramduth D Thobakgale C Chetty S Rathnavalu P Moore C Pfafferott KJ Hilton L Dominant influence of HLA-B in mediating the potential co-evolution of HIV and HLA Nature 2004 432 769 775 10.1038/nature03113 15592417

[B27] Salazar-Gonzalez JF Bailes E Pham KT Salazar MG Guffey MB Keele BF Derdeyn CA Farmer P Hunter E Allen S Deciphering human immunodeficiency virus type 1 transmission and early envelope diversification by single-genome amplification and sequencing J Virol 2008 82 3952 3970 10.1128/JVI.02660-07 18256145 PMC2293010

[B28] Thompson JD Higgins DG Gibson TJ CLUSTAL W: improving the sensitivity of progressive multiple sequence alignment through sequence weighting, position-specific gap penalties and weight matrix choice Nucleic Acids Res 1994 22 4673 4680 10.1093/nar/22.22.4673 7984417 PMC308517

[B29] Posada D Crandall KA MODELTEST: testing the model of DNA substitution Bioinformatics 1998 14 817 818 10.1093/bioinformatics/14.9.817 9918953

[B30] Rambaut A FigTree v1.1.2 2008 http://tree.bio.ed.ac.uk/software/figtree

[B31] Drummond A Rambaut A BEAST: Bayesian evolutionary analysis by sampling trees BMC Evol Biol 2007 7 214 10.1186/1471-2148-7-214 17996036 PMC2247476

[B32] Tamura K Dudley J Nei M Kumar S MEGA4: Molecular Evolutionary Genetics Analysis (MEGA) software version 4.0 Mol Biol Evol 2007 24 1596 1599 10.1093/molbev/msm092 17488738

[B33] Korber B Myers G Signature pattern analysis: a method for assessing viral sequence relatedness AIDS Res Hum Retroviruses 1992 8 1549 1560 10.1089/aid.1992.8.1549 1457200

[B34] Yang Z PAML: a program package for phylogenetic analysis by maximum likelihood Comput Appl Biosci 1997 13 555 556 9367129 10.1093/bioinformatics/13.5.555

[B35] Pond SL Frost SD Muse SV HyPhy: hypothesis testing using phylogenies Bioinformatics 2005 21 676 679 10.1093/bioinformatics/bti079 15509596

[B36] Diskin R Marcovecchio PM Bjorkman PJ Structure of a clade C HIV-1 gp120 bound to CD4 and CD4-induced antibody reveals anti-CD4 polyreactivity Nat Struct Mol Biol 17 608 613 10.1038/nsmb.1796 20357769 PMC2949298

[B37] Schwede T Kopp J Guex N Peitsch MC SWISS-MODEL: An automated protein homology-modeling server Nucleic Acids Res 2003 31 3381 3385 10.1093/nar/gkg520 12824332 PMC168927

[B38] Weissenhorn W Dessen A Harrison SC Skehel JJ Wiley DC Atomic structure of the ectodomain from HIV-1 gp41 Nature 1997 387 426 430 10.1038/387426a0 9163431

[B39] DeLano W The PyMOL Molecular Graphics System 2006 2006 DeLano Scientific, San Carlos, CA, USA

[B40] Sagar M Wu X Lee S Overbaugh J Human immunodeficiency virus type 1 V1-V2 envelope loop sequences expand and add glycosylation sites over the course of infection, and these modifications affect antibody neutralization sensitivity J Virol 2006 80 9586 9598 10.1128/JVI.00141-06 16973562 PMC1617272

[B41] Huang W Toma J Fransen S Stawiski E Reeves JD Whitcomb JM Parkin N Petropoulos CJ Coreceptor tropism can be influenced by amino acid substitutions in the gp41 transmembrane subunit of human immunodeficiency virus type 1 envelope protein J Virol 2008 82 5584 5593 10.1128/JVI.02676-07 18353956 PMC2395220

[B42] Yang W Bielawski JP Yang Z Widespread adaptive evolution in the human immunodeficiency virus type 1 genome J Mol Evol 2003 57 212 221 10.1007/s00239-003-2467-9 14562964

[B43] Zwick MB Jensen R Church S Wang M Stiegler G Kunert R Katinger H Burton DR Anti-human immunodeficiency virus type 1 (HIV-1) antibodies 2F5 and 4E10 require surprisingly few crucial residues in the membrane-proximal external region of glycoprotein gp41 to neutralize HIV-1 J Virol 2005 79 1252 1261 10.1128/JVI.79.2.1252-1261.2005 15613352 PMC538539

[B44] Mani I Gilbert P Sankale JL Eisen G Mboup S Kanki PJ Intrapatient diversity and its correlation with viral setpoint in human immunodeficiency virus type 1 CRF02_A/G-IbNG infection J Virol 2002 76 10745 10755 10.1128/JVI.76.21.10745-10755.2002 12368317 PMC136616

[B45] McNearney T Hornickova Z Markham R Birdwell A Arens M Saah A Ratner L Relationship of human immunodeficiency virus type 1 sequence heterogeneity to stage of disease Proc Natl Acad Sci USA 1992 89 10247 10251 10.1073/pnas.89.21.10247 1438212 PMC50315

[B46] Markham RB Wang WC Weisstein AE Wang Z Munoz A Templeton A Margolick J Vlahov D Quinn T Farzadegan H Yu XF Patterns of HIV-1 evolution in individuals with differing rates of CD4 T cell decline Proc Natl Acad Sci USA 1998 95 12568 12573 10.1073/pnas.95.21.12568 9770526 PMC22871

[B47] Gray ES Moore PL Choge IA Decker JM Bibollet-Ruche F Li H Leseka N Treurnicht F Mlisana K Shaw GM Neutralizing antibody responses in acute human immunodeficiency virus type 1 subtype C infection J Virol 2007 81 6187 6196 10.1128/JVI.00239-07 17409164 PMC1900112

[B48] Menzo S Sampaolesi R Vicenzi E Santagostino E Liuzzi G Chirianni A Piazza M Cohen OJ Bagnarelli P Clementi M Rare mutations in a domain crucial for V3-loop structure prevail in replicating HIV from long-term non-progressors Aids 1998 12 985 997 10.1097/00002030-199809000-00004 9662194

[B49] Gaschen B Taylor J Yusim K Foley B Gao F Lang D Novitsky V Haynes B Hahn BH Bhattacharya T Korber B Diversity considerations in HIV-1 vaccine selection Science 2002 296 2354 2360 10.1126/science.1070441 12089434

[B50] Derdeyn CA Decker JM Bibollet-Ruche F Mokili JL Muldoon M Denham SA Heil ML Kasolo F Musonda R Hahn BH Envelope-constrained neutralization-sensitive HIV-1 after heterosexual transmission Science 2004 303 2019 2022 10.1126/science.1093137 15044802

[B51] Rong R Bibollet-Ruche F Mulenga J Allen S Blackwell JL Derdeyn CA Role of V1V2 and other human immunodeficiency virus type 1 envelope domains in resistance to autologous neutralization during clade C infection J Virol 2007 81 1350 1359 10.1128/JVI.01839-06 17079307 PMC1797511

[B52] Liu Y Curlin ME Diem K Zhao H Ghosh AK Zhu H Woodward AS Maenza J Stevens CE Stekler J Env length and N-linked glycosylation following transmission of human immunodeficiency virus Type 1 subtype B viruses Virology 2008 374 229 233 10.1016/j.virol.2008.01.029 18314154 PMC2441482

[B53] Lynch RM Shen T Gnanakaran S Derdeyn CA Appreciating HIV type 1 diversity: subtype differences in Env AIDS Res Hum Retroviruses 2009 25 237 248 10.1089/aid.2008.0219 19327047 PMC2853864

[B54] Gnanakaran S Lang D Daniels M Bhattacharya T Derdeyn CA Korber B Clade-specific differences between human immunodeficiency virus type 1 clades B and C: diversity and correlations in C3-V4 regions of gp120 J Virol 2007 81 4886 4891 10.1128/JVI.01954-06 17166900 PMC1900169

